# The Clinical Significance of Extrapancreatic Incidental Findings Detected During Pancreas Surveillance

**DOI:** 10.1016/j.gastha.2025.100818

**Published:** 2025-09-27

**Authors:** Helena Saba, Elizabeth Abou Diwan, Hassan Sinan, Dimitri Melki, Mohamad Dbouk, Amanda Blackford, Linda Chu, Eun Ji Shin, Marcia Irene Canto, Michael Goggins

**Affiliations:** 1 Department of Pathology, The Sol Goldman Pancreatic Cancer Research Center, Johns Hopkins Medical Institutions, Baltimore, Maryland; 2 Department of Oncology, The Sol Goldman Pancreatic Cancer Research Center, Johns Hopkins Medical Institutions, Baltimore, Maryland; 3 Department of Radiology, The Sol Goldman Pancreatic Cancer Research Center, Johns Hopkins Medical Institutions, Baltimore, Maryland; 4 Department of Medicine, The Sol Goldman Pancreatic Cancer Research Center, Johns Hopkins Medical Institutions, Baltimore, Maryland

**Keywords:** cancer screening, incidentalomas, overdiagnosis, overtreatment

## Abstract

**Background and Aims:**

Cancer screening tests have the potential to detect cancers and precancerous conditions, but one concern is the potential that screen-detected incidental findings lead to unnecessary, potentially harmful interventions. The aim of this study is to determine, the yield of extrapancreatic incidental findings that required management, including cancers, precancerous lesions, and other findings of diagnostic concern in patients undergoing pancreatic surveillance for their familial or genetic pancreatic cancer risk.

**Methods:**

We reviewed the pancreatic surveillance records of 925 patients enrolled in the Cancer of Pancreas Screening studies at The Johns Hopkins Hospital from 1998 to 2023 who had undergone at least one pancreatic surveillance test. Significant extra-pancreatic incidental findings that required diagnostic evaluation and/or treatment were tabulated, including the resulting diagnostic tests, therapeutic interventions, and complications.

**Results:**

The mean age/standard deviation at enrollment, of patients in the cohort was 58.0/10.4 years; 59.7% female), 220 (23.8%) had undergone baseline surveillance, 705 (76.2%) had undergone surveillance for a median of 4 years. Seventy-five extrapancreatic incidental findings were detected in 68 patients (7.3%), including 10 cancers, 80% of which were stage I and 8 (0.86%) precancerous lesions. Thirteen patients underwent surgical (n = 13), endoscopic (n = 9), or chemotherapy (n = 4). Twenty-five patients (2.7%) were recommended to undergo surveillance, (20 for nondysplastic Barrett’s esophagus). Three patients (0.33%) were considered overtreated in retrospect (2 cholecystectomies, 1 partial nephrectomy, all laparoscopic). One patient had a postoperative complication treated successfully.

**Conclusion:**

Extrapancreatic incidental findings are commonly detected in patients undergoing pancreatic surveillance, but few signify serious pathology. Almost all interventions for incidental findings were for cancerous and precancerous lesions, with few instances of unnecessary interventions.

## Introduction

Recent studies, based on the accumulation of ∼25 years of experience, have demonstrated that pancreatic surveillance of high-risk individuals can result in down-staging of pancreatic cancers diagnosed, with the majority of screen-detected pancreatic cancers diagnosed at stage I disease, and long-term survival in most patients.^1^, ^2^, ^3^, ^4^, ^5^

Many individuals who undergo pancreatic surveillance are at increased risk for other cancers. The tests used for surveillance endoscopic ultrasound (EUS) and magnetic resonance imaging (MRI)/magnetic retrograde cholangiopancreatography,^6^, ^7^, ^8^ not only have the potential to detect other cancers, they often uncover incidental findings.

The diagnostic yield of EUS and MRI for pancreatic lesions in patients undergoing pancreatic surveillance has been extensively studied,^9^, ^10^, ^11^, ^12^, ^13^, ^14^ but the significance of nonpancreatic incidental findings identified during pancreatic surveillance has not been extensively studied.^15^^,^^16^ The conundrum of detecting incidental findings is that their significance is often uncertain without additional workup.^17^, ^18^, ^19^ Occasionally, the evaluation of incidental findings results in significant physical, emotional, and/or financial burden, including complications arising from their diagnostic workup and/or therapeutic intervention. On the other hand, timely detection of significant incidental findings such as early-stage cancers has the potential to be life-saving.

The aim of this study was to determine the diagnostic yield and summarize the subsequent outcome of incidental findings requiring management in a large cohort undergoing pancreatic surveillance.

## Methods

### Study Design and Patients

We analyzed prospectively collected and medical record data from patients enrolled in the Cancer of Pancreas Screening studies (CAPS) at The Johns Hopkins Hospital from 1998 to March 2023. The CAPS cohort has been described elsewhere.^1^^,^^6^^,^^7^ The CAPS studies (NA_00087754, NA_00044490, NA_00005455) were approved by the Johns Hopkins Institutional Review Board and informed consent was obtained from all prospectively enrolled patients.

Patients in the CAPS cohort undergo pancreatic surveillance with EUS or MRI for their family history of pancreatic cancer and/or for germline mutation status per the CAPS international consensus guidelines.^6^^,^^7^ If indicated, an upper endoscopy with biopsy is performed at the time of EUS. Pancreatic protocol computed tomography (CT) is no longer used as a first-line surveillance test for most patients but was evaluated as a screening test in the early years of pancreatic surveillance. For this study, individuals had to have had at least a baseline pancreatic screening imaging test (EUS, MRI, or CT). Our final cohort was composed of 925 patients.

Information about incidental findings was retrieved by reviewing relevant imaging reports, pathology reports, laboratory reports, and physicians’ notes. We also reviewed our CAPS REDCap questionnaire data, sent annually to patients, that asks about newly diagnosed pancreatic and extrapancreatic malignancies. We confirmed patient-documented diagnoses with their electronic medical record.

We recorded any abnormal extrapancreatic findings found during pancreatic surveillance. Tests done to evaluate symptoms or abnormal laboratory results (including new-onset diabetes mellitus), or to further characterize a previously known extrapancreatic lesion not first detected through pancreatic surveillance were not included in the analysis.

We limited our analysis to extrapancreatic incidental findings that warranted diagnostic and/or therapeutic intervention, including but not limited to, additional imaging (for example, MRI, CT/PET, ultrasonography/endoscopic evaluation, etc.), consultative referrals, biopsies, medication/chemotherapy administration, and surgical management. Thus, commonly detected incidental findings that did not require further evaluation or treatment (such as simple cysts of the liver, kidney or ovary, asymptomatic gallstones, hemangiomas, small adrenal nodules, mild gastritis, small gastric/duodenal erosions, evidence of esophagitis, and hiatal hernias) were not tabulated unless there were notable features that warranted additional clinical evaluation. We also did not tabulate changes in the management of common endoscopic findings such as treatment for reflux esophagitis or gastritis, or other common findings that may have led to further evaluation and treatment, such as hepatic steatosis. (In a separate analysis, we found hepatic steatosis documented in 24.7% of 900 CAPS patients undergoing EUS surveillance).

Extrapancreatic incidental findings were classified into 3 categories: 1) malignant: (such as renal cell carcinoma (RCC)); 2) premalignant (e.g. Barrett’s esophagus with dysplasia); and 3) non-neoplastic: The latter included Barrett’s esophagus without dysplasia, and other imaging findings that ultimately were determined to be benign (eg lymphadenopathy).

Some patients in the cohort developed cancers arising from symptoms or other screening tests, not by pancreatic surveillance; we only tabulated cancers detected by pancreatic surveillance tests.

We classified diagnostic interventions into one of 2 types; (1) invasive (eg biopsy or fine needle aspiration during EUS, colonoscopy, percutaneous liver biopsy, cystoscopy, etc.) and (2) noninvasive (eg, imaging tests, laboratory blood workup, etc.).

Similarly, we classified therapeutic interventions into 1 of 3 categories: 1) invasive (eg, open partial nephrectomy), 2) minimally invasive (eg laparoscopic cholecystectomy, endoscopic cryoballoon ablation), and 3) other (eg medications/chemotherapy).

### Statistical Analysis

Continuous variables were summarized with means and standard deviations whereas categorical variables were summarized as absolute and relative frequencies. All analyses were performed using IBM SPSS® Statistics Data Software. The primary study endpoints were the number and stage of extrapancreatic cancers and premalignant conditions and the number of incidental findings that led to a diagnostic workup and therapeutic intervention, and whether such intervention was worthwhile, or unnecessary. We also compared the age-adjusted incidence of renal cell cancer (ICD-O-3 code C64.9) to the expected incidence in our cohort, matching on age, sex, and race using SEER∗Stat version 8.4.1.

## Results

The baseline characteristics of the 925 study subjects are presented in Table 1. The majority of patients were female (59.7%), White (93.6%), and non-Hispanic or Latino (97.9%). The mean patient age at the start of surveillance was 58 years (standard deviation; 10.4 years). Sixty-four percent of patients (596) were undergoing surveillance because of their family history of pancreatic cancer, 329 (35.6%) patients had a known pathogenic germline variant (with or without family history), including 149 (45.3%) with a *BRCA2*, 44 (13.8%) with an *ATM*, and 45 (13.7%) with a *BRCA1* variant.

**Table 1:** Characteristics of Participants Undergoing Pancreas Surveillance

	Total n = 925 (%)
Demographics	
Age at first visit	
Mean (SD)	58.0 (10.4)
IQR	50–65
Sex	
Female	552 (59.7)
Male	373 (40.3)
Race	
White	866 (93.6)
Black	32 (3.5)
Asian	13 (1.4)
Other	11 (1.2)
Unknown	3 (0.3)
Ethnicity	
Non-hispanic or latino	906 (97.9)
Hispanic or latino	11 (1.2)
Other	2 (0.2)
Unknown	6 (0.6)
Smoking status	
Current smoker	31 (3.6)
Former smoker	271 (29.3)
Indication for surveillance	
Family history of pancreatic cancer^a^	596 (64.4)
Mutation carriers (including those with a family history of pancreatic cancer)	
BRCA2	149 (16.1)
BRCA1	45 (4.9)
ATM	44 (4.8)
PALB2	30 (3.2)
CDKN2A	26 (2.8)
Lynch syndrome	22 (2.4)
Peutz-Jeghers syndrome (STK11)	13 (1.4)

a at least one first- and one second-degree relative with pancreatic cancer on the same side of the family.

Of the 925 subjects, 705 (76.2%) had at least 2 screening visits; 220 (23.8%) had baseline imaging only. The remaining 705 (76.2%) high-risk individuals had a median follow-up duration of 4 years (interquartile range, 1.58–8.25 years).

Overall, 45 (4.9%) patients underwent surgical resection for surveillance-detected pancreatic lesions; 14 for pancreatic cancer, 6 had high-grade dysplasia within pancreatic intraepithelial neoplasm or intraductal papillary mucinous neoplasm, and 3 had a pancreatic neuroendocrine tumor; the remaining cases had pancreatic intraepithelial neoplasia (PanIN) and/or intraductal papillary mucinous neoplasm with low-grade dysplasia. A detailed description of these pancreatic findings and their outcomes has been reported previously.^1^^,^^4^^,^^20^

Seventy-five notable nonpancreatic incidental findings that required additional diagnostic evaluation and/or management were identified in 68 patients (7.3% of cohort). Thirty-four of the 75 (45.3%) incidental findings were detected by EUS/EGD, 29 (38.7%) by MRI, and 12 (16%) by CT.

### Extrapancreatic Malignancies

Extrapancreatic malignancies were diagnosed as a direct result of pancreatic imaging tests in 1.1% of the cohort (10 of 925) (Table 2). These 10 cases are only a subset of the (n = 45) extrapancreatic cancers diagnosed in this cohort during the study period.^1^ Eight of the 10 extrapancreatic malignancies detected were stage I. Of the 10 cancers detected as a result of pancreatic imaging, 5 were RCCs; all 5 were stage I. We determined that the age-adjusted incidence rate of RCC in our cohort was not significantly different from the incidence in SEER (data not shown). The other 3 stage I cancers included a patient with an esophageal adenocarcinoma, another with a transitional cell carcinoma of the bladder, and one with chronic lymphocytic leukemia.

**Table 2:** Significant Nonpancreatic Neoplasms Detected Incidentally During Pancreas Surveillance

Pt no.	Age/Gender	Risk group	Incidental Finding	Imaging test	Additional diagnostic tests	Referrals	Final pathological diagnosis	Tumor stage	Management
Cancers									
1	53/M	FPC	Renal mass 3 cm	CT	None	Urology	RCC	T1a N0 M0	Laparoscopic radical nephrectomy
2	65/M	FPC	Renal mass 2.3 cm	MRI	CT	Urology	RCC	T1a N0 M0	Open partial nephrectomy^a^
3	71/M	FPC	Renal mass 1 cm	MRI	CT	Urology	RCC	T1a N0 M0	Laparoscopic partial nephrectomy
4	59/F	FPC	Renal lesion 6 mm	MRI	CT	Urology	RCC	T1a N0 M0	Open partial nephrectomy
5	52/M	BRCA2	Renal mass 2.2 cm	MRI	CT	Urology	RCC	T1a N0 M0	Laparoscopic partial nephrectomy
7	72/M	FPC Former 0.4 pack year smoker	Bladder base mass	CT	Cystoscopy with biopsy	Urology/Oncology	TCC of the bladder	T1a N0 M0	TURBT, chemotherapy
8	51/F	FPC	Mesenteric lymphadenopathy	CT	EUS with biopsy	Hem/Onc	B Cell CLL	Stage I	Chemotherapy
9	60/M	FPC	7 hypoechoic peripancreatic lesions, largest 1 cm	EUS	FNA	Hem/Onc	B Cell lymphoma	Stage IV	Chemotherapy
10	57/F	FPC	1 cm polyp at GEJ	EUS/EGD	Biopsy	None	Intramucosal adenocarcinoma with Barrett’s with HGD	T1a N0 M0	EMR, endoscopic cryoablation
54	76 F	FPC	3m cystic/solid right ovary mass	CT	None	Gynecology	Ovarian cancer	Stage 3	TAHBSO^b^ and chemoradiation
Precancerous lesions									
11	50/M	BRCA2	Suspected Barrett’s esophagus	EUS/EGD	Biopsy	None	Barrett’s esophagus with HGD	N/A	Endoscopic cryoablation
12	36/F	FPC	Appendiceal mass 5.6 cm	MRI	CT	General surgery	Low-grade appendiceal mucinous neoplasm	Tis^c^ M0	Open appendectomy, ileocecectomy
13	87/F	FPC	Suspected appendiceal mass	MRI	CT	General surgery	Low-grade appendiceal mucinous neoplasm	Tis^c^ M0	Laparoscopic appendectomy, partial ileocecectomy
14	65/M	FPC	1 cm gastric polyp	EUS/EGD	Biopsy	None	Gastric neuroendocrine tumor in the background of autoimmune gastritis	Stage I	ESD
15	61/M	Lynch syndrome	1 cm Flat duodenal polyp	EGD	Biopsy	None	Duodenal tubular adenoma	N/A	Endoscopic polypectomy
6	73/M	FPC	Renal lesion 1.3 cm	MRI	CT	Urology	Renal papillary adenoma	N/A	Laparoscopic partial nephrectomy
7	80/M	FPC	Flat duodenal polyp 1 cm	EGD	Biopsy	None	Duodenal tubular adenoma	N/A	Endoscopic polypectomy + cryoablation
55	43/F	PJS	Flat duodenal polyp 0.5 cm	EGD	Biopsy	None	Duodenal tubular adenoma	N/A	Endoscopic polypectomy

All patients were subsequently surveilled following standard of care guidelines for their malignancies or premalignancies.

CLL, Chronic lymphocytic leukemia; EMR, endoscopic mucosal resection; ESD, endoscopic submucosal dissection; FNA, fine needle aspiration; FPC, familial pancreatic cancer; HGD, high-grade dysplasia; GEJ, Gastroesophageal junction; PJS, Peutz-Jegher Syndrome; TAHBSO, total abdominal hysterectomy with bilateral salpingo-oophorectomy; TCC, transitional cell carcinoma; TURBT, Transurethral resection of bladder tumor.

a Complicated by a pseudoaneurysm requiring coiled embolization 14 d postsurgery.

b Total abdominal hysterectomy with bilateral salpingo-oophorectomy.

c In-situ.

One patient had stage IV B cell lymphoma that was treated with chemotherapy, and 1 patient had stage III ovarian cancer and underwent chemotherapy after surgical resection. All 10 patients are alive at the last follow-up without evidence of recurrent disease (Table 2).

### Premalignant Lesions

Eight patients had a premalignant lesion diagnosed and treated (including tubular duodenal adenoma, renal papillary adenoma, low-grade appendiceal mucinous neoplasm, gastric neuroendocrine tumor, Barrett esophagus with high-grade dysplasia, and renal papillary adenoma) (see Table 2).

In addition to the 2 patients with Barrett’s and esophageal adenocarcinoma and/or high-grade dysplasia, another 20 patients were diagnosed with nondysplastic Barrett’s esophagus (Table 3).

**Table 3:** Extrapancreatic Gastrointestinal Non-neoplastic Incidentalomas Requiring Investigation

Incidental findings (#)	Pt#	Description	Imaging Test	Referral	Diagnostic intervention	Diagnosis	Management
Barrett’s esophagus (20)	7, 16–26, 61–68	Suspected Barrett’s with erosion	EUS	N/A	Biopsy	Nondysplastic Barrett’s esophagus	Surveillance [for BE]
Gallbladder abnormalities (4)	27	0.5 cm polyps	MRI	General surgery	US	Cholesterol polyps	Lap^a^ cholecystectomy
	28	1.4 cm nodule	EUS	General surgery	MRI	Chronic cholecystitis	Lap^a^ cholecystectomy
	29	1.3 cm multilocular lesion	MRI	N/A	US	Focal adenomyomatosis	None
	58	Cyst with dilation of common bile duct	EUS	N/A	ERCP	Choledochal cyst	Surveillance
Liver abnormalities (3)	30	2.8 cm ill-defined lesion	MRI	Hepatology	Liver biopsy, CT, lab workup	Benign indeterminate nodule	Surveillance [for liver nodule]
	31	6.6 cm cyst	MRI	Hepatology	MRI	Benign simple cyst	None
	32	0.7 cm subcapsular lesion	CT	Hepatology	MRI, lab workup	Hemangioma	None
Gastric abnormalities (4)	33	2.2 cm mass, possibly a lipoma	MRI	N/A	EUS with biopsy	Benign indeterminate lesion	None
	56	1.5 polyp in body and fundus	EUS	N/A	EUS with biopsy	Hamartoma in the setting of Peutz-Jegher syndrome	Endoscopic polypectomy
	57	0.4 cm polyp in body	EUS	N/A	EUS with biopsy	Gastric fundic gland polyps	Endoscopic polypectomy
	54	0.3 erythematous polyp in antrum	EUS	N/A	EUS with biopsy	Inflammatory polyp in setting of *H.Pylori* with Focal intestinal metaplasia	Endoscopic polypectomy
Esophagitis^b^	34	Fine, whitish exudates in mid-esophagus	EUS	N/A	Biopsy	Candida esophagitis	Antifungals
Duodenal mass	35	2.2 cm air- and debris-containing mass	MRI	N/A	EUS/duodenoscopy	Periampullary air-filled diverticulum	None
Peripancreatic LN	36	4 peripancreatic, hypoechoic, heterogenous LN measuring up to 1.7 mm	EUS	N/A	Fine needle biopsy	Benign LN	None
Possible mesenteric mass	37	Suspicious soft tissue density	MRI	N/A	CT	Normal	None
Colonic narrowing	38	Circumferential 2 cm mid transverse colonic narrowing	MRI	N/A	Colonoscopy	Normal colon	None
Foreign body	39	3 cm linear metallic density anterior to the descending colon	CT	General surgery	N/A	Metal shard^c^	Lap^a^ foreign body removal

BE, Barrett’s esophagus; ERCP, endoscopic retrograde cholangiopancreatography; LN, lymph node; US, ultrasound.

a Laparoscopic.

b Infectious only.

c Retained catheter fragment from previous diagnostic laparoscopy in the setting of ovarian torsion.

### Other Benign Incidentalomas

Fifty-seven non-neoplastic extrapancreatic lesions were detected that warranted additional diagnostic evaluation and/or therapeutic intervention, 37 within the gastrointestinal tract (Table 3), 20 outside the gastrointestinal tract (Table 4). Gallbladder (4), liver (3), and gastric abnormalities (4), were the most common sites of gastrointestinal tract abnormalities. Among the nongastrointestinal incidentalomas, lung nodules (5), aneurysmal dilation (4), and ovarian cysts (3) were the main findings.

**Table 4:** Extrapancreatic Nongastrointestinal Incidentalomas Requiring Investigation

Incidental findings (no^a^.)	Pt no.^a^	Description	Imaging Test	Referral	Diagnostic intervention^b^	Diagnosis^c^	Management
Lung nodule (5)	34	1.2 cm lingular lung lesion	MRI	N/A	Low-dose CT chest	Atelectasis	None
	8	4 enlarging nodules (<0.5 cm)	CT	Pulmonary	Low-dose CT chest	Benign nodule	None
	40	Indeterminate 1.3 cm nodule	MRI	Pulmonary	Low-dose CT chest	Benign nodule	None
	41	0.4 cm nodule	MRI	N/A	Low-dose CT chest	Benign nodule	None
	42^d^	0.5 cm bilateral scattered nodules, concerning for metastasis	CT^d^	Pulmonary	Low-dose CT chest	Benign nodules	None
Aneurysmal dilatation (4)	34	4.6 cm aneurysmal dilatation of ascending aorta	CT	Cardiology	Echocardiogram, thoracoabdominal CT, CTA chest	Ascending aortic aneurysm	Surveillance [for aneurysm]
	43	4.4 cm ectatic ascending thoracic aorta	CT	N/A	CTA chest	Ascending aortic aneurysm	Surveillance [for aneurysm]
	44	0.9 cm aneurysmal dilatation of SMA branch	MRI	Vascular	Abdominal arterial duplex US	SMA-branch aneurysm	Surveillance [for aneurysm]
	45	3.7 cm transverse aneurysmal dilatation of suprahepatic IVC	MRI	Vascular, cardiology	Thoracoabdominal CT	Suprahepatic IVC aneurysm	Surveillance [for aneurysm]
Complex ovarian cyst (3)	46	3 cm hyperintense focus	MRI	Gynecology	Pelvic US	Benign cyst	None
	47	7 cm septated cyst	CT	Gynecology	Pelvic US	Dystrophic calcification	None
	59	3 cm T2 hyperintense focus in left adnexa	MRI	Gynecology	Pelvic US	Normal ovarian cyst	None
Renal lesion (2)	48	Indeterminate 1.6 cm exophytic hypodensity with low attenuation	CT	N/A	Renal CT	Benign cyst	None
	61	Multiple non enhancing large renal cysts (largest 10 cm on right)	MRI	Urology	Renal CT	Polycystic kidney disease	Surveillance
Unilateral hydrouretero-nephrosis (2)	49	New right hydroureteronephrosis	MRI	Urology	CT urogram	UPJ and distal ureteral calculus	Retrograde ureteric stent placement
	50	Mild right hydronephrosis and hydroureter	MRI	PCP	Renal CT	No obstructive mass	None
Breast cysts (2)	51	Bilateral hypoechoic cystic lesions	MRI	Gynecology	Breast US, Mammogram	Fibrocystic changes	None
	60	1.5 × 1.2 cm hyperintense lesion of the left breast	MRI	Gynecology	Breast US, Mammogram	Simple cyst - aspirated	None
Vertebral bone lesions (2)	52	Enhancing indeterminate 1.3 cm T10 vertebral body lesion	MRI	PCP	PET scan	Lytic bone lesion	Per PCP
	53	Diffuse enhancement of L4 vertebral body	MRI	PCP	Lumbar spine MRI	Benign/possible Paget's disease	Per PCP

CTA, Computed tomography angiography; IVC, inferior vena cava; PCP, Primary care physician; PET, Positron emission tomography; SMA, superior mesenteric artery; UPJ, Ureteropelvic junction; US, ultrasound.

a Number.

b No minimally invasive or invasive intervention was done in this subgroup.

c Clinical or pathological when available.

d Progressor in screening (HG-PanIN), CT, done status postdistal pancreatectomy.

Twenty-five patients were recommended to undergo surveillance (including the 20 with non-dysplastic Barrett’s), 8 patients received endoscopic (n = 3), surgical (n = 4) or medical therapy (n = 1). After additional diagnostic tests, twenty of these 57 incidental findings were determined to not require further surveillance or treatment.

### Overtreatment/Complications

Seventeen patients without cancer required therapeutic interventions for surveillance-detected incidental findings, most of which were minimally invasive procedures (Tables 2 and 3). In retrospect, three of these patients can be considered as overtreated. Two of these patients underwent laparoscopic cholecystectomy, one for a gall bladder polyp that had increased in size from 5 mm to 8 mm-it turned out to be a cholesterol polyp, and one patient for a 1.2 cm gallbladder polyp caused by adenomyomatosis (patients #27 and #28, Table 3). One patient underwent a laparoscopic partial nephrectomy for what turned out to be a renal papillary adenoma (patient #6, Table 2).

Only one patient had a postoperative complication related to treatment of an incidentaloma; this patient had a pseudoaneurysm successfully treated by a coil placed by interventional radiology 14 days following an open partial nephrectomy for renal cell cancer.

## Discussion

An inevitable outcome of clinical imaging tests is the detection of unrelated incidental findings. This is especially the case for patients undergoing cancer screening or surveillance as these patients undergo regular imaging, increasing the likelihood of detecting incidental findings.

The yield of extra-pancreatic incidentalomas detected during pancreas surveillance has been subjected to limited reporting.^15^^,^^16^ Unlike prior studies, we limited our analysis to incidental findings that required further management. Sawhney and colleagues reported on the prevalence of incidentalomas in 180 patients, 60% of whom were BRCA1/2 mutation carriers.^16^ Ibrahim et al.^15^ described incidental findings in three European cohorts totaling 568 subjects ∼40% of whom were *CKDN2A-Leiden* mutation carriers. In this European study, MRI was the main surveillance test used in 2 of the 3 cohorts. Sawhney and colleagues reported more incidentalomas, including significant incidentalomas, detected with MRI than with EUS.^16^ In our study, 8 of the 10 extra-pancreatic cancers and 3 of the precancerous lesions were identified by MRI/CT. In our cohort, almost two-thirds of patients were under surveillance for their familial risk, 9 of the 10 cancers occurred in the familial risk group, and all but 2 of them were Stage I. In contrast, the majority of the cancers reported by Ibrahim et al. (7 of 11, 63.6%) were observed in the *CKDN2A* mutation carriers, and most of the cancers were detected at advanced stage. Extra-pancreatic cancers were detected in 2.2% of patients by Sawhney and colleagues; tumor stage was not reported.^16^ The cancer yield in our study was 1.1%. It was 1.9% in the European study, which also had a somewhat longer median duration of surveillance/follow-up than our study (4.7 years).

In our study, we limited our analysis to incidentalomas that required further diagnostic workup or therapeutic intervention. Although MRI and EUS are considered complementary pancreatic surveillance tests overall, one advantage of EUS, along with concurrent EGD over MRI is its greater ability to visualize the upper gastrointestinal tract. Among the lesions identified by EUS/EGD, 2 significant findings, a stage I esophageal cancer and a high-grade dysplasia within Barrett’s would likely not have been detected at the same early-disease stage by MRI. On the other hand, several extragastrointestinal cancers would not have been detected by EUS/EGD, including the 5 renal cancers and 2 pelvic cancers which were detected by CT prior to 2010 when CT was still being evaluated as a primary pancreatic surveillance test.^14^ Several patients were identified as having aortic aneurysms and referred for ongoing surveillance. A USPSTF guideline recommends one-time screening for male ever-smokers because of their increased risk and the reduction in aortic aneurysm-related mortality that can be achieved with ongoing surveillance and management.^21^ EGD also enabled the detection of Barrett’s esophagus in 2.4% of the cohort. These 20 patients found to have Barrett’s esophagus do not include 13 additional patients known to have Barrett’s esophagus at the start of their pancreatic surveillance. EUS-based pancreatic surveillance not only facilitates detection of Barrett’s, ongoing Barrett’s surveillance can be done in conjunction with regular EUS. Indeed, 1 patient is being followed for Barrett’s with low-grade dysplasia, having been treated with endoscopic cryoballoon ablation. Some patients have concurrent indications for upper endoscopic surveillance, such as those with Lynch syndrome and Peutz-Jegher syndrome. Three of our patients had duodenal adenomas detected during surveillance. Carriers of cancer susceptibility gene mutations that increase pancreatic cancer risk also increase the risk of periampullary cancers (cholangiocarcinoma, ampullary and duodenal cancers) that can be detected during pancreatic surveillance with EUS and MRI.^22^ Although our study was not designed to formally compare the diagnostic yield of different surveillance strategies, our results highlight the complementary diagnostic yield of alternating MRI/magnetic retrograde cholangiopancreatography and EUS.

Some extrapancreatic findings such as fatty liver (also known as hepatic steatosis or metabolic dysfunction–associated steatotic liver disease [MASLD]) are highly prevalent and readily identified by MRI and EUS. The detection of MASLD not only has management implications not only because it can cause advanced liver disease, and is associated with cardiovascular disease, diabetes and other metabolic conditions,^23^^,^^24^ it has also recently emerged as a risk factor for pancreatic,^25^, ^26^, ^27^ and other cancers.^27^ We have also identified MASLD as a risk factor for pancreatic cancer among patients undergoing pancreas surveillance (Sinan et al, unpublished). While metabolic risk factors such as MASLD are not currently incorporated into decisions regarding pancreatic surveillance, they could be in the future, once the cancer risk related to MASLD is better understood, including its relation to disease severity, etiology, duration, etc.

While some incidental findings might signify serious pathology, others are pathologically inconsequential but can still be a source of anxiety, testing and overtreatment. Several studies have evaluated the problem of surgical overtreatment of screen-detected pancreatic lesions,^28^^,^^29^ but the assessment of overtreatment of extrapancreatic incidental findings detected during pancreatic surveillance is mostly lacking. Ibrahim et al. reported that less than 1% (0.9%, 5 of 568) of their cohort underwent surgery for a benign lesion, which compares similarly to the 0.33% yield described in our study (3 of 925)(2 laparoscopic cholecystectomies and a laparoscopic partial nephrectomy). The resection of a gastric neuroendocrine tumor arising from autoimmune gastritis could also represent overtreatment. However, while many small neuroendocrine tumors have a benign course, it is difficult to predict the natural course of individual neuroendocrine tumors. Overall, the rate of overtreatment of benign incidental findings in our cohort was low and no patients suffered a complication related to investigation of a benign extrapancreatic incidental finding.

Another aspect of potential overtreatment of screen-detected lesions is the problem of cancer overdiagnosis. While cancer screening tests should enhance the balance of benefits and harms,^30^ in practice, some cancers discovered incidentally would not have caused harm, or come to clinical attention during an individual’s natural lifespan, if not for the screening. Overdiagnosis can lead to a rapid rise in cancer incidence, but without a corresponding increase in mortality,^31^ and is a feature of cancer types with a high proportion of indolent cancers (ie thyroid, prostate, renal, breast cancer, and melanoma).^32^ It is possible that some of the RCCs diagnosed in our cohort represent overdiagnosis,^33^ though patients with germline mutation often have aggressive cancers. For example, patients with pathogenic *BRCA2* variants (such as one of our RCC cases) have an increased risk of renal cancer progression.^34^ Patients in the CAPS program are counseled about their other cancer surveillance according to evidence-based guidelines individualized for patient’s germline mutation status and family history. Germline mutation carriers require personalized surveillance recommendations tailored to their main cancer risks.^35^

Understanding the incidence of significant incidentalomas found during pancreas surveillance can help clinicians better understand the risk/benefits of surveillance and can help them tailor pancreas surveillance modalities to those that best suit individual patients.

Some strengths of our study include the large sample size of our cohort. Some limitations of this study include that it is a retrospective, single-center study of a predominantly White cohort. Disparities in enrollment into pancreatic surveillance programs has been highlighted in prior studies.^36^^,^^37^

## Conclusion

Most patients that had incidental findings detected during their pancreatic surveillance likely benefited from their detection, though few represent serious pathology and fewer still were overtreated.
